# Cardiac ultrasomics for acute myocardial infarction risk stratification and prediction of all-cause mortality: a feasibility study

**DOI:** 10.1186/s44156-024-00057-w

**Published:** 2024-09-16

**Authors:** Quincy A. Hathaway, Ankush D. Jamthikar, Nivedita Rajiv, Bernard R. Chaitman, Jeffrey L. Carson, Naveena Yanamala, Partho P. Sengupta

**Affiliations:** 1 Division of Cardiovascular Disease and Hypertension, Department of Medicine, Rutgers Robert Wood Johnson Medical School, New Brunswick, NJ USA; 2 Department of Radiology, University of Pennsylvania, Philadelphia, PA USA; 3 Department of Medicine, St. Louis University School of Medicine, St. Louis, MO USA; 4 Division of General Internal Medicine, Department of Medicine, Rutgers Robert Wood Johnson Medical School, New Brunswick, NJ USA; 5 Rutgers Robert Wood Johnson Medical School, Division of Cardiovascular Disease and Hypertension, 125 Patterson St, New Brunswick, NJ 08901 USA

**Keywords:** Topology, TDA, Semantic segmentation, Ultrasomics, Machine learning

## Abstract

**Background:**

Current risk stratification tools for acute myocardial infarction (AMI) have limitations, particularly in predicting mortality. This study utilizes cardiac ultrasound radiomics (i.e., ultrasomics) to risk stratify AMI patients when predicting all-cause mortality.

**Results:**

The study included 197 patients: (a) retrospective internal cohort (*n* = 155) of non-ST-elevation myocardial infarction (*n* = 63) and ST-elevation myocardial infarction (*n* = 92) patients, and (b) external cohort from the multicenter Door-To-Unload in ST-segment–elevation myocardial infarction [DTU-STEMI] Pilot Trial (*n* = 42). Echocardiography images of apical 2, 3, and 4-chamber were processed through an automated deep-learning pipeline to extract ultrasomic features. Unsupervised machine learning (topological data analysis) generated AMI clusters followed by a supervised classifier to generate individual predicted probabilities. Validation included assessing the incremental value of predicted probabilities over the Global Registry of Acute Coronary Events (GRACE) risk score 2.0 to predict 1-year all-cause mortality in the internal cohort and infarct size in the external cohort. Three phenogroups were identified: Cluster A (high-risk), Cluster B (intermediate-risk), and Cluster C (low-risk). Cluster A patients had decreased LV ejection fraction (*P* < 0.01) and global longitudinal strain (*P* = 0.03) and increased mortality at 1-year (log rank *P* = 0.05). Ultrasomics features alone (C-Index: 0.74 vs. 0.70, *P* = 0.04) and combined with global longitudinal strain (C-Index: 0.81 vs. 0.70, *P* < 0.01) increased prediction of mortality beyond the GRACE 2.0 score. In the DTU-STEMI clinical trial, Cluster A was associated with larger infarct size (> 10% LV mass, *P* < 0.01), compared to remaining clusters.

**Conclusions:**

Ultrasomics-based phenogroup clustering, augmented by TDA and supervised machine learning, provides a novel approach for AMI risk stratification.

**Supplementary Information:**

The online version contains supplementary material available at 10.1186/s44156-024-00057-w.

## Background

Globally, acute myocardial infarction (AMI) affects nearly 10% of people over 60 years of age [1]. In the United States, the total annual cost of AMI was $85 billion in 2016, with an estimated $40 billion lost due to premature mortality in the preceding decade [2]. Unfortunately, despite the success of intervention and evolving guideline-directed treatment, AMI patients continue to have high morbidity and mortality [3]. Currently, clinicians use validated risk stratification scoring systems, such as the Global Registry of Acute Coronary Events (GRACE) [4, 5] and more recently the GRACE 2.0 score [6], to predict the 6-month and 1-year risk of all-cause mortality following AMI. While guidelines have recommended using the GRACE score as the most robust model for all acute coronary syndrome types [7–9], these scores were developed using clinical trial data long before percutaneous interventions became routine. Moreover, GRACE uses conventional statistical approaches (i.e., logistic regression) with fixed linear assumptions on data behavior and limited variables, resulting in modest discrimination (e.g., C-statistic range for predicting mortality:0.65–0.8) [5, 9].

Artificial intelligence (AI) techniques have led to the development of novel methods that includes subjecting images and other inputs to sophisticated algorithms to capture complexity of human health and disease at the level of the individual [10]. These methods have achieved remarkable success, especially in disease classification and risk assessments, in several image-based disciplines, such as dermatology, gastroenterology, ophthalmology, oncology, and neuroradiology [10–16], including the development of ‘omics’-based decision support tools [17–21]. The application of radiomics to cardiac ultrasound (i.e., ultrasomics), may aid in risk stratification of patients experiencing an AMI by extracting texture-based information from the myocardium. Moreover, the development of automated tools that integrate ultrasomics for AMI risk stratification addresses the existing gap in current guidelines which do not currently integrate cardiac imaging-based information in existing tools like GRACE 2.0 for estimating risk.

In the present study, we used a cluster-then-predict approach for AMI risk stratification. We subjected cardiac ultrasomics information to topological data analysis (TDA)—a robust method to create compressed representations of highly dimensional data to create unique patient phenogroups [22]. We illustrate that the ultrasomics phenogroups can provide independent and incremental information to conventional tools like GRACE 2.0 for augmenting 1-year mortality prediction in AMI patients. Moreover, TDA can be effectively combined with machine learning and explainable AI techniques. Accordingly, we also illustrate the ability to develop robust supervised machine-learning algorithms on clustered patients, which can be applied to external data for phenogroup prediction. Since infract size is strongly associated with all-cause mortality in AMI [23], we used the Door-To-Unload in STEMI (DTU-STEMI) Pilot Trial [24] as an external, prospective, multicenter clinical trial cohort to illustrate that the high-risk phenogroup had larger infarct size as observed on cardiac magnetic resonance (CMR) imaging.

## Methods

### Study population

**Fig. 1:**
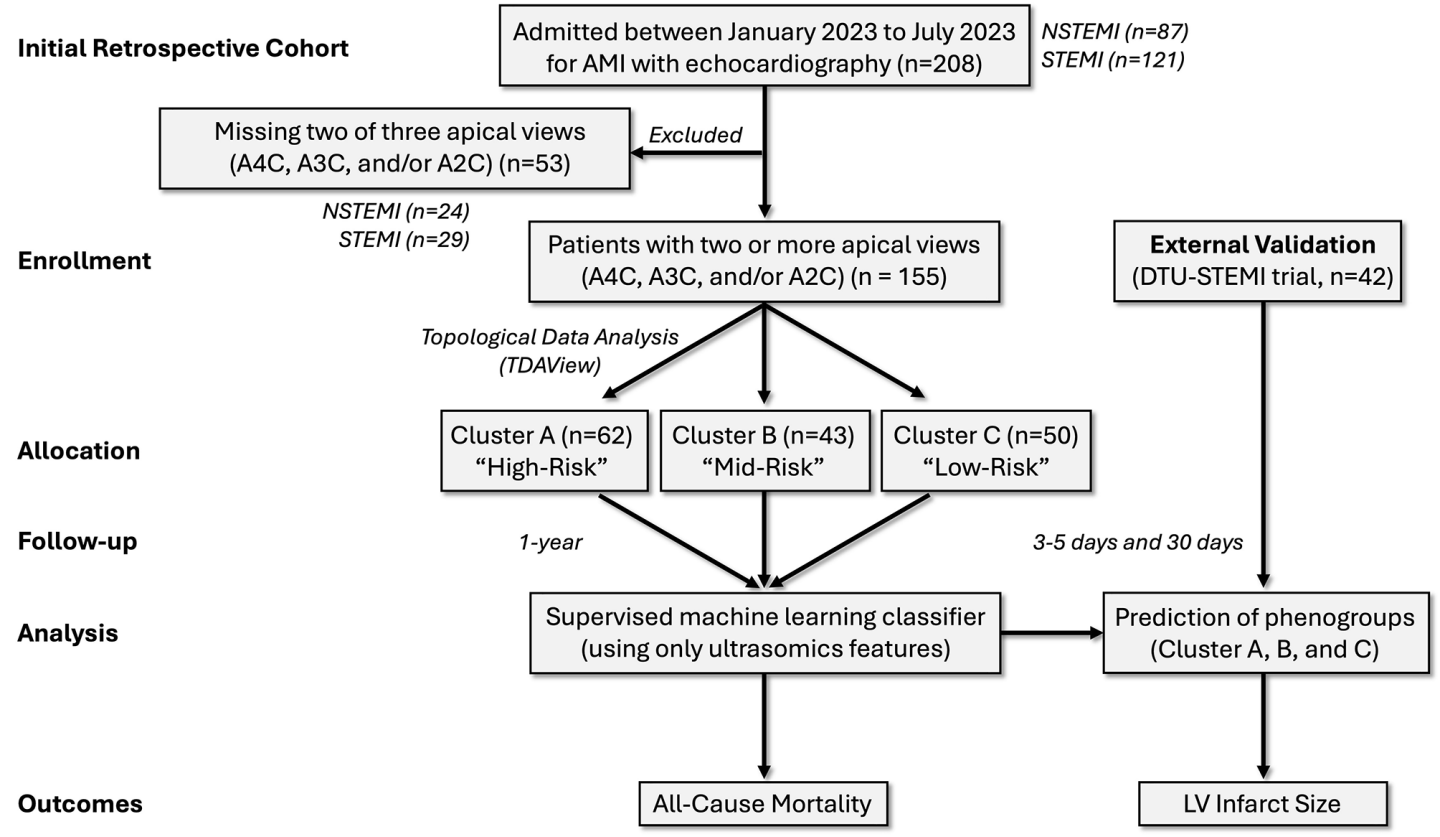
Recruitment Diagram. Patients (*n* = 208) were retrospectively identified over a 6 month timeline who were admitted for AMI. Of these patients, 155 patients were included in the study who had at least two of three apical echocardiographic views available for analysis. Using ultrasomics features from the images, topological data analysis was used to cluster patients into three groups. These three groups were assessed in a supervised machine learning algorithm to develop class labels for the external validation group. Ultimately, groups clustered using ultrasomics features were assessed for prediction of all-cause mortality and left ventricular infarct size

For the internal validation dataset, AMI patients were retrospectively identified from the electronic medical record of Robert Wood Johnson University Hospital who were admitted over a 6-month period between January 2023 to July 2023 (Fig. 1). The Institutional Review Board (IRB) of Robert Wood Johnson University Hospital gave ethical approval for this work (#Pro2023001660). STEMI was classified per the Joint ESC/ACCF/AHA/WHF Task Force [25]. Exclusion criteria included [1] patients discharged to institutionalized care [2], type 2–5 AMI [3], co-existing terminal illness with palliative care for cancer, neurological illness (severe dementia, motor neuron disease, multiple sclerosis, Parkinson’s disease, stroke, supranuclear palsy and multiple system atrophy), heart, lung, kidney or liver failure [4] alternative diagnosis for elevated cardiac troponin values (e.g. myocarditis, pericarditis, non-ischemic cardiomyopathies, moderate-severe valvular heart disease, sepsis, aortic dissection, blunt cardiac injury, coronary spasm and vasculitis, arrhythmia and cardiac arrest), and [5] pregnancy. After applying the exclusion criteria, 208 patients were initially enrolled (i.e., 87 patients classified as having a non-ST-elevation myocardial infarction (NSTEMI) and 121 as having a ST-elevation myocardial infarction (STEMI)). Of the 208 patients initially enrolled, 53 patients were further excluded from analysis due to technically insufficient imaging for 2 of the following 3 views: apical 4 chamber (A4C), apical 3 chamber (A3C), and apical 2 chamber (A2C). Technically insufficient imaging was classified as an inability to delineate the left ventricle (LV) endocardial boundaries on visual inspection for 2 or more segments. After excluding patients without at least two of the three apical views, 155 patients were identified for subsequent analysis (including 63 patients classified as having a NSTEMI and 92 as having a STEMI). We assessed the performance of the GRACE 2.0 score [6] with the primary outcome of all-cause mortality at one year.

For the external validation dataset, participants were recruited from a prospective, multicenter, randomized Door-To-Unload in ST-segment–elevation myocardial infarction (DTU-STEMI) pilot trial [24] (Fig. 1). We included 42 participants (all participants classified as having a STEMI) with CMR data in the current study. Infarct size on CMR was used as the primary end point. CMR-quantified infarct size was categorized as large (LGE mass accounting for > 10% of the total LV mass) or small (LGE mass accounts for ≤ 10% of the total LV mass) [26, 27]. The details of the CMR protocol have been previously described [24]. Briefly, patients in the DTU-STEMI trial underwent standard CMR with steady-state free-precession sequence for LV ejection fraction, volumes, and mass analysis on days 3 to 5 and again on day 30 (± 7 days). For the external cohort, institutional review boards at each site approved the trial, and patients provided written, informed consent. The study was approved by the Food and Drug Administration (NCT03000270, Registration Date: 12/12/2016, Last Update: 05/06/2019).

### Echocardiography image acquisition, preprocessing, and semantic segmentation

Echocardiograms from A4C, A3C, and A2C were utilized in the present studies for both the internal and external validation data analysis. Patients and participants required at least two of the three views to be present to be included in the current study (*see Materials*, section* Study Population*). 2D echocardiograms were preprocessed from video formats to DICOM using Sante DICOM Viewer Pro (SanteSoft, Nicosia, Cyprus, Greece). DICOM files containing doppler data, dual ultrasound regions, or other with limited technical views were discarded. A4C, A3C, and A2C multi-beat echocardiogram DICOM files were manually selected. The LV was segmented in the A4C, A3C, and A2C views using echocv [28] (i.e., a semantic segmentation algorithm that automatically defines regions of the heart in echocardiography images through convolutional neural networks (CNNs)).

Echocv and its validation has previously been published [28], we modified echocv to be executed using Python 3.2 and leveraged TensorFlow 1.15.0 with GPU support, alongside CUDA 10.0. The segmented images were also uniformly resized to a fixed shape of 1024 by 1024 to ensure consistency across various image sources. Otherwise the use of algorithm and its validation has previously been published, specifically for predicting LV remodeling in parasternal long axis echocardiograms [29]. Using the semantic segmentation algorithm, a binary mask representing the region of interest (ROI) within the A4C, A3C, and A2C views was achieved (Figure S1A). The ROI for each of the three views was then processed to obtained radiomics/ultrasomics-based information.

### First-Order, shape, and texture-based feature extraction

Echocardiography ultrasomics were extracted in Python (v3.7.13) using pyradiomics (v3.0.1) [30], SimpleITK (v2.2.0) [31], pywavelets (v1.3.0), and numpy (v1.21.5) for both the internal and external validation sets. We have previously published using this methodology on the LV [29]. Briefly, feature extraction was performed for the 2D ROI using featureextractor() from pyradiomics. Default parameters for extraction, binwidth, resampled pixel spacing, interpolator, label definition, were applied. In total, first-order (*n* = 18), shape (*n* = 9), and texture-based (*n* = 73) features were extracted for each of the echocardiography views (i.e., A4C, A3C, and A2C) (Figure S1B).

### TDA

The online tool TDAView [32] was used for phenogroup cluster of AMI patients in the internal validation set. Briefly, TDAView utilizes the Mapper algorithm based on TDAmapper [33]. This includes user defined variables for Mapper such as: filter function, number of intervals, proportion of overlap, and number of bins in single-linkage clustering. The Mapper function allows geometric information to be converted into high dimensional point cloud data that can be interpreted by varying filters [33]. Our goal with the current work was to delineate AMI patients with “high-risk” features from those with “low-risk” features when predicting all-cause mortality. By decreasing the number of bins and the range of the lens values (i.e., intervals), we can effectively decrease the amount of oversampling and number of edges created from the resultant clusters. We used a 1D Mapper filter with distance function as Euclidean and filter function as mean. Number of intervals was defined as 10, with 5 bins. To reduce the overlap between clusters, a 5% overlap was defined. The number of clusters was not fixed. Based on the parameters used in TDAView, three clusters were generated, labeled as Cluster A (*n* = 62), B (*n* = 43), and C (*n* = 50).

### Supervised machine learning classifier

BigML (https://bigml.com. BigML, Inc. Corvallis, Oregon, USA) was utilized for supervised machine learning and to develop a classifier for prediction of patients in Cluster A, B, and C. Weights were applied to Cluster A (weight = 1), Cluster B (weight = 1.189), and Cluster C (weight = 1.023) to address class imbalance. Through the OptiML application (i.e., a supervised machine learning algorithm that compares generated ensembles, deep neural networks, and logistic regression algorithms) 10-fold cross validation was performed and prediction of Cluster A, B, and C phenogroups was performed using only ultrasomics features. Once the supervised classifier was developed, the external validation set (*n* = 42 participants) was analyzed by the model to generate predicted class labels. These class labels (i.e., Cluster A, B, and C) were used for subsequent outcome prediction.

### Statistics

GraphPad Prism (v10.1.1) and R (v4.1.0) were used for statistical analyses. The Shapiro-Wilk test assessed normality. In normally distributed data with continuous variables, a two-sided Student’s t-test was applied. In non-Gaussian distributed data, the Mann-Whitney test was used. When assessing more than one group of continuous variables, a one-way analysis of variance (ANOVA) was applied. A Dunnett’s multiple comparisons test was used for multiple comparisons in the one-way ANOVA. When assessing more than one group of categorical variables, a non-parametric Kruskal-Wallis test was applied with multiple comparisons testing.

Receiver operating characteristics (ROC) area under the curve (AUC) was created using the BigML platform, utilizing 10-fold cross validation. A Kaplan-Meier curve was generated using the R packages survival (v3.4-0) [34] and survminer (v0.4.9). Stratification of events, assessed as patients at risk for mortality at one year, was performed over 50-day increments for patients in Cluster A, Cluster B, and Cluster C. The *P*-value was calculated using the log-rank test in R. Using the survival package, a Cox Proportional Hazard model (CoxPH) for time-to-event analyses of mortality at one year was assessed. A risk score was generated with the (A) GRACE 2.0 score alone, (B) GRACE + Cluster A, (C) GRACE + LV global longitudinal strain, and (D) using all three variables through CoxPH regression. A probability score (i.e., ranging from 0 to 1) for predicting outcomes was generated using the predictRisk function of the riskRegression (v2022.11.28) package in R. The concordance index (C-statistic) was calculated using the pec (v2022.05.04) package in R [35].

## Results

### Study overview

**Fig. 2:**
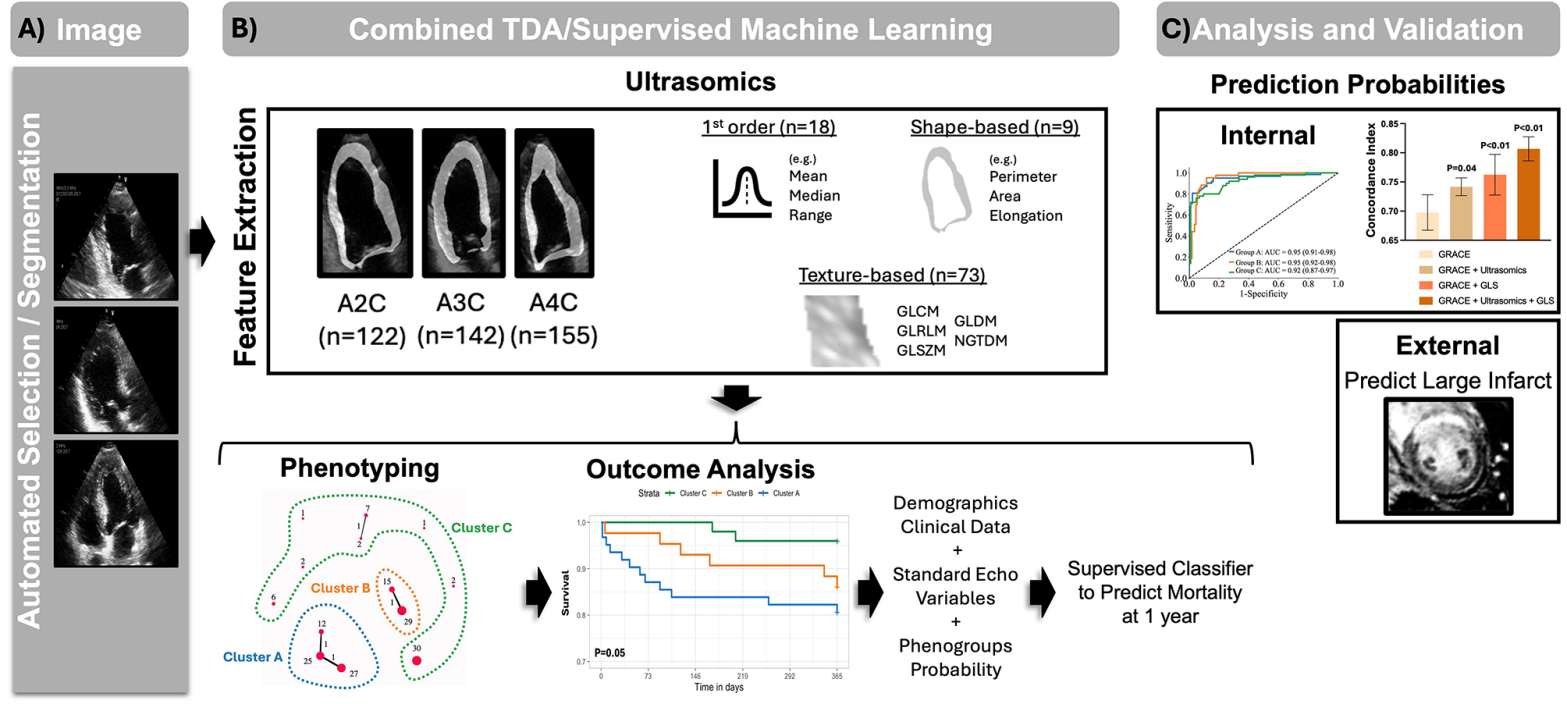
Study Design and Overview. (**A**) The internal validation patient cohort presenting with non-ST-elevation myocardial infarction (NSTEMI, *n* = 63) and ST-elevation myocardial infarction (STEMI, *n* = 92) (**B**) Ultrasomics features were extracted and TDAView was used to cluster patients into three phenogroups: Cluster A, Cluster B, and Cluster C. The identified phenogroups were used to develop class labels for the external validation group using a supervised classifier. (**C**) The generated probabilities from the supervised classifier were used to predict mortality and illustrate the incremental value of ultrasomics features over GRACE 2.0. The supervised classifier was applied to the external validation group to develop class labels, which were used to predict findings on cardiac magnetic resonance, including acute infarct size

We evaluated patients (*n* = 155) presenting with NSTEMI and STEMI who had at least two of three apical echocardiographic views acquired during admission (Fig. 2A). Using echocardiography-derived ultrasomics, phenogroups were labeled through TDA and applied to the prediction of clinical outcomes, such as time-to-event mortality (Fig. 2B). A supervised machine learning algorithm was further used to characterize which ultrasomics features are important in prediction of the phenogroups and generation of risk prediction score. We then evaluated the incremental value of the phenogroups using the internal validation group and explored how assigned phenogroup labels contributed to predicting CMR findings in the external validation group (Fig. 2C).

### Patient demographics and functional parameters – internal validation

Demographic features for patients in the internal validation study presenting with NSTEMI (*n* = 63) and STEMI (*n* = 92) were assessed (Table 1). Patients presenting with NSTEMI were less likely to have a history of congestive heart failure (CHF) (1.59% vs. 20.65%, *P* < 0.01) and lower GRACE Score (107.92 vs. 120.63, *P* = 0.02), compared to STEMI patients, respectively. Patients presenting with NSTEMI were more likely to have a history of coronary artery disease (CAD) (52.38% vs. 19.57%, *P* < 0.01), chonic kindey disease (CKD) (23.81% vs. 10.87%, *P* = 0.03), and stroke (17.46% vs. 6.52%, *P* = 0.03), compared to STEMI patients, respectively. When comparing the groups based on type of AMI, there were no differences in outcomes, including major adverse cardiac events (MACE) at 30 days (*P* = 0.38), cardiovascular death at 1 year (*P* = 0.89), and all-cause mortality at 1 year (*P* = 0.95).

**Table 1:** Patient demographics of the Internal Validation Group Stratified by Acute myocardial infarction (AMI). Patients presenting with non-ST-elevation myocardial infarction (NSTEMI, *n* = 63) and ST-elevation myocardial infarction (STEMI, *n* = 92). Data are presented as the percent (%) of total or the 95% confidence interval, where applicable. Data are considered statistically significant if *P* ≤ 0.05, denoted by * and bolded text. BMI = body mass index, CHF = congestive heart failure, COPD = chronic obstructive pulmonary disease, CAD = coronary artery disease, CKD = chronic kidney disease, GRACE = Global Registry of Acute coronary events, MACE = major adverse cardiac events

Internal Validation - Patient Demographics Stratified by Acute Myocardial Infarction (AMI)			
Variable	NSTEMI (*n* = 63)	STEMI (*n* = 92)	*P*-Value
Age (years)	68.03 (66.48–69.58)	65.47 (64.04–66.9)	0.28
Sex (Male)	40 (63.49%)	70 (76.09%)	0.09
Race/Ethnicity Caucasian Asian American Hispanic American Black/African American	24 (38.1%) 8 (12.7%) 14 (22.22%) 6 (9.52%)	37 (40.22%) 22 (23.91%) 14 (15.22%) 8 (8.70%)	0.79 0.08 0.27 0.86
BMI (kg/m^2^)	27.82 (27.09–28.55)	28.43 (27.42–29.44)	0.67
Systolic Blood Pressure (mmHg)	143 (140–146)	143 (140–147)	0.96
Diastolic Blood Pressure (mmHg)	74 (72–75)	80 (78–82)	0.05
Heart Rate (per minute)	84 (81–86)	85 (83–87)	0.66
Cardiac Arrest (at admission)	0 (0%)	4 (4.35%)	0.09
Troponin Elevation (at admission)	63 (100%)	89 (96.74%)	0.15
Smoking History Current Former	11 (17.46%) 18 (28.57%)	18 (19.57%) 22 (24.18%)	0.74 0.54
History of CHF	1 (1.59%)	19 (20.65%)	*<0.01
History of COPD	5 (7.94%)	2 (2.17%)	0.09
History of CAD	33 (52.38%)	18 (19.57%)	*<0.01
History of CKD	15 (23.81%)	10 (10.87%)	*0.03
History of Diabetes Mellitus	35 (55.56%)	39 (42.39%)	0.11
History of Hyperlipidemia	38 (60.32%)	51 (55.43%)	0.55
Prior Myocardial Infarction	12 (19.05%)	13 (14.29%)	0.43
Prior Percutaneous Intervention	22 (34.92%)	25 (27.17%)	0.31
Prior Coronary Artery Bypass Graft	7 (11.11%)	7 (7.61%)	0.46
Prior Stroke	11 (17.46%)	6 (6.52%)	*0.03
GRACE Score	107.92 (105.04–110.8)	120.63 (116.97-124.28)	*0.02
MACE at 30 Days	6 (9.52%)	13 (14.29%)	0.38
Cardiovascular Death − 1 year	5 (8.06%)	8 (8.70%)	0.89
All-Cause Mortality − 1 year	8 (12.70%)	12 (13.04%)	0.95

Echocardiographic functional features for patients in the internal validation study presenting with NSTEMI (*n* = 63) and STEMI (*n* = 92) were assessed (Table 2). Patients presenting with STEMI were more likely to have a reduced LV ejection fraction (48% vs. 53%, *P* < 0.01) and left atrial end-systolic volume index (23 mL/m^2^ vs. 29 mL/m^2^, *P* < 0.01), compared to NSTEMI patients, respectively. Further the LV wall motion score index (2.00 vs. 1.70, *P* < 0.01) and LV global longitudinal strain (-11.86 vs. -14.10, *P* < 0.01) indicated greater wall motion abnormalities in STEMI compared to NSTEMI patients, respectively.

**Table 2:** Patient cardiac function of the Internal Validation Group Stratified by Acute myocardial infarction (AMI). Patients presenting with non-ST-elevation myocardial infarction (NSTEMI, *n* = 63) and ST-elevation myocardial infarction (STEMI, *n* = 92). Data are presented as the percent (%) of total or the 95% confidence interval, where applicable. Data are considered statistically significant if *P* ≤ 0.05, denoted by * and bolded text

Internal Validation - Patient Cardiac Function Stratified by Acute Myocardial Infarction (AMI)			
Variable	NSTEMI (*n* = 63)	STEMI (*n* = 92)	*P*-Value
Left Ventricular Internal Diameter - End Diastole (mm)	46 [45–47]	47 [45–49]	0.38
Left Ventricular Internal Diameter - End Systole (mm)	34 [32–36]	37 [35–39]	0.07
Left Ventricular Mass Index (g/m²)	87 (81–93)	92 (85–98)	0.35
Left Ventricular End-diastole Volume (mL)	94 (86–103)	106 (99–113)	0.06
Left Ventricular End-systole Volume (mL)	47 [40–53]	57 (51–62)	*0.03
Left Ventricular Ejection Fraction (%)	53 (50–56)	48 [45–50]	*<0.01
Left Ventricular Wall Motion Score Index	1.70 (1.56–1.83)	2.00 (1.90–2.11)	*<0.01
Left Ventricular Global Longitudinal Strain (%)	-14.10 (-15.07- -13.12)	-11.86 (-12.64- -11.08)	*<0.01
Left Ventricular Outflow Tract Stroke Volume (mL)	61 (56–66)	55 (51–59)	0.12
e’ Septal	5.90 (5.47–6.33)	6.04 (5.64–6.43)	0.64
e’ Lateral	8.26 (7.51–9.02)	7.79 (7.26–8.32)	0.95
Mitral Valve E Wave (cm/s)	85 (78–91)	83 (77–89)	0.81
MV-A (cm/s)	85 (79–91)	79 (74–84)	0.21
E/A Ratio	1.06 (0.94–1.18)	1.05 (0.96–1.14)	0.92
E/e’ Septal	15.70 (13.71–17.69)	15.06 (13.66–16.45)	0.64
E/e’ Lateral	11.57 (10.19–12.94)	11.63 (10.44–12.82)	0.95
Left Atrial End-systolic Volume Index (mL/m^2^)	29 [26–31]	23 [21–25]	*<0.01

### Phenogroup Clustering through TDA

**Fig. 3:**
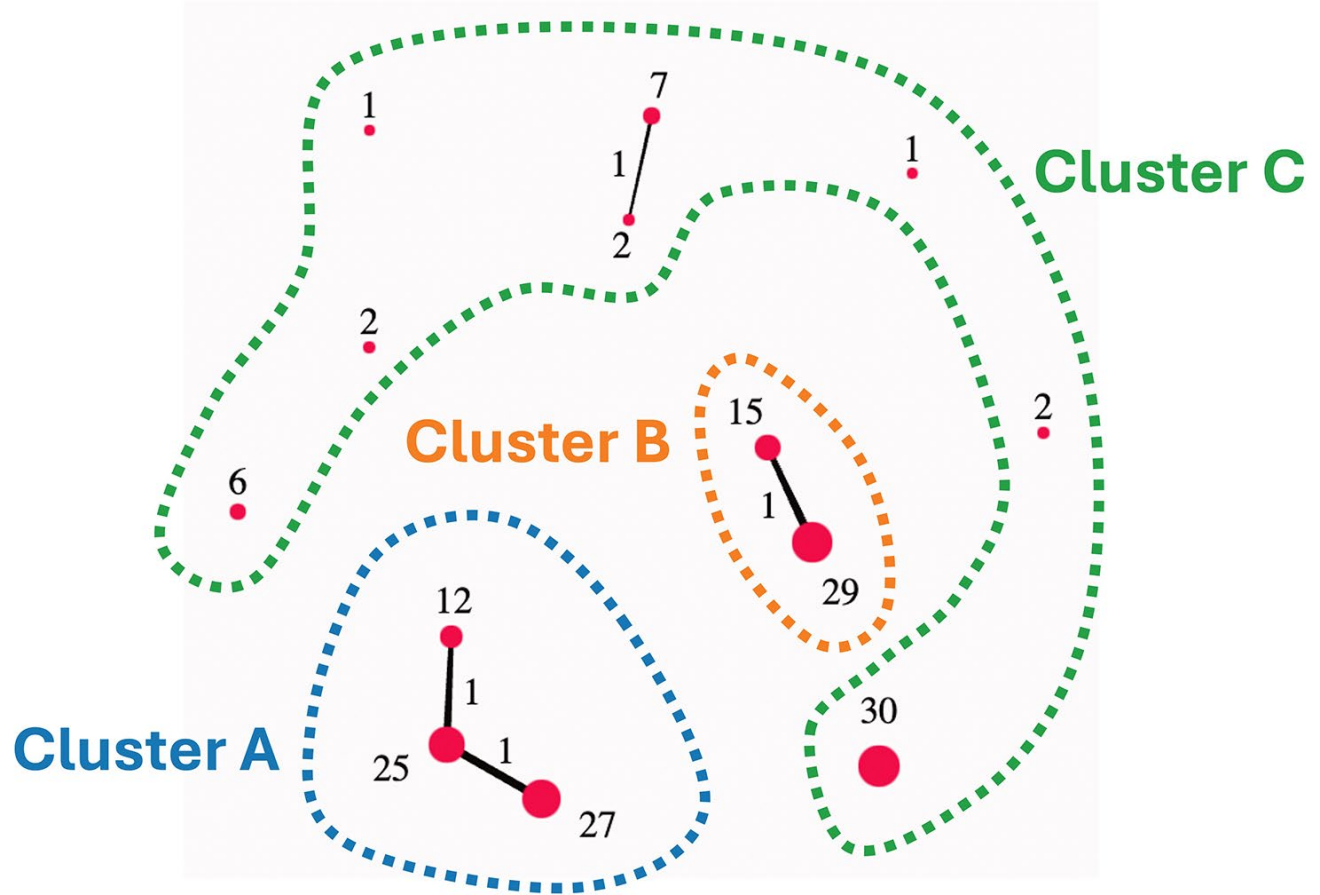
Topological Data Analysis (TDA) Clustering of Ultrasomics Features. Individual nodes are represented as red circles, with the number next to the node corresponding to the number of patients included in the node. Cluster A (*n* = 62), Cluster B (*n* = 43), and Cluster C (*n* = 50)

Using the online tool TDAView, three phenogroups were identified: Cluster A (*n* = 62), Cluster B (*n* = 43), and Cluster C (*n* = 50) (Fig. 3). Of these phenogroups, Cluster A and Cluster B are illustrated to be more homogenous in their connectivity within groups, whereas Cluster C is illustrated to represent a more heterogenous compilation of patients. Assessing the differences between these clusters, Cluster A contains more patients with a prior history of CHF (22.58% vs. 8.00%, *P* = 0.04), compared to Cluster C (Table 3). Further, the Cluster A phenogroup has a higher risk of all-cause mortality at 1 year (19.35% vs. 4.00%, *P* = 0.03), compared to Cluster C. The data in Table 2 highlight how the Cluster A represents a “high-risk” phenogroup, whereas Cluster B can be seen as “intermediate-risk” and Cluster C as “low-risk”. When assessing the echocardiographic functional parameters (Table 4), Cluster A had a reduced LV ejection fraction (45% vs. 53%, *P* < 0.01) and LV global longitudinal strain (-11.88 vs. -13.87, *P* = 0.03) compared to Cluster C, respectively.

**Table 3:** Patient demographics of the Internal Validation Group for Predicted Ultrasomics Phenogroups. Using only the ultrasomics features from the A4C, A3C, and A2C echocardiogram views, patients were clustered into phenogroups. Cluster a “high-risk” (*n* = 62), cluster B “intermediate-risk” (*n* = 43), and cluster C “low-risk” (*n* = 50) using topological data analysis (TDA). Data are presented as the percent (%) of total or the 95% confidence interval, where applicable. Data are considered statistically significant if *P* ≤ 0.05, denoted by * and bolded text. BMI = body mass index, CHF = congestive heart failure, COPD = chronic obstructive pulmonary disease, CAD = coronary artery disease, CKD = chronic kidney disease, STEMI = ST-elevation myocardial infarction, GRACE = Global Registry of Acute coronary events, MACE = major adverse cardiac events

Internal Validation - Patient Demographics in Predicted Ultrasomics Phenogroups				
Variable	Cluster A (High Risk) (*n* = 62)	Cluster B (*n* = 43)	Cluster C (Low Risk) (*n* = 50)	*P*-Value
Age (years)	66.74 (62.98–70.51)	66.88 (62.34–71.43)	65.9 (62.03–69.77)	0.94
Sex (Male)	44 (70.97%)	31 (72.09%)	35 (70.00%)	0.98
Race/Ethnicity Caucasian Asian American Hispanic American Black/African American	24 (38.71%) 12 (19.35%) 9 (14.52%) 5 (8.07%)	16 (37.21%) 8 (18.6%) 9 (20.93%) 4 (9.30%)	21 (42.00%) 10 (20.00%) 10 (20.00%) 5 (10.00%)	0.89 0.99 0.64 0.94
BMI (kg/m^2^)	29.01 (26.08–31.93)	28.9 (26.91–30.89)	26.56 (24.68–28.43)	0.28
Systolic Blood Pressure (mmHg)	140 (132–149)	145 (135–155)	145 (136–155)	0.65
Diastolic Blood Pressure (mmHg)	78 (73–84)	77 (71–84)	76 (71–80)	0.72
Heart Rate (per minute)	86 (81–92)	85 (78–93)	81 (76–87)	0.47
Cardiac Arrest (at admission)	2 (3.23%)	1 (2.33%)	1 (2.00%)	0.92
Troponin Elevation (at admission)	61 (98.39%)	43 (100%)	48 (96.00%)	0.37
STEMI (at admission)	36 (58.06%)	26 (60.47%)	30 (60.00%)	0.96
Smoking History Current Former	16 (25.81%) 10 (16.13%)	11 (25.58%) 6 (13.95%)	13 (26.53%) 13 (26.00%)	0.99 0.27
History of CHF	14 (22.58%)**	2 (4.65%)	4 (8.00%)	*0.01
History of COPD	1 (1.61%)	4 (9.30%)	2 (4.00%)	0.17
History of CAD	21 (33.87%)	18 (41.86%)	12 (24.00%)	0.19
History of CKD	10 (16.13%)	5 (11.63%)	10 (20.00%)	0.55
History of Diabetes Mellitus	30 (48.39%)	19 (44.19%)	25 (50.00%)	0.85
History of Hyperlipidemia	34 (54.84%)	25 (58.14%)	30 (60.00%)	0.86
Prior Myocardial Infarction	8 (12.90%)	10 (23.26%)	7 (14.29%)	0.34
Prior Percutaneous Intervention	5 (8.07%)	5 (11.63%)	4 (8.00%)	0.67
Prior Coronary Artery Bypass Graft	21 (33.87%)	13 (30.23%)	13 (26.00%)	0.79
Prior Stroke	6 (9.68%)	4 (9.302%)	7 (14.00%)	0.71
GRACE Score	118.1 (109.1-127.2)	114.5 (104.8-124.3)	112.8 (103.9-121.8)	0.69
MACE at 30 Days	7 (11.29%)	5 (11.63%)	7 (14.29%)	0.88
Cardiovascular Death − 1 year	8 (13.11%)	4 (9.30%)	1 (2.00%)	0.11
All-Cause Mortality − 1 year	12 (19.35%)**	6 (13.95%)	2 (4.00%)	*0.04

* = *P* ≤ 0.05 when comparing all groups

** = *P* ≤ 0.05 when comparing Cluster A vs. Cluster C

**Table 4:** Patient cardiac function of the Internal Validation Group for Predicted Ultrasomics Phenogroups. Using only the ultrasomics features from the A4C, A3C, and A2C echocardiogram views, patients were clustered into phenogroups. Cluster a “high-risk” (*n* = 62), cluster B “intermediate-risk” (*n* = 43), and cluster C “low-risk” (*n* = 50) using topological data analysis (TDA). Data are presented as the percent (%) of total or the 95% confidence interval, where applicable. Data are considered statistically significant if *P* ≤ 0.05, denoted by * and bolded text

Internal Validation - Patient Cardiac Function in Predicted Ultrasomics Phenogroups				
Variable	Cluster A (High Risk) (*n* = 62)	Cluster B (*n* = 43)	Cluster C (Low Risk) (*n* = 50)	*P*-Value
Left Ventricular Internal Diameter - End Diastole (mm)	48 [46–50]	46 [43–49]	45 [43–47]	0.17
Left Ventricular Internal Diameter - End Systole (mm)	37 [35–40]**	35 [31–38]	33 [31–36]	*0.04
Left Ventricular Mass Index (g/m²)	92 (84–99)	85 (76–93)	91 (81–101)	0.53
Left Ventricular End-diastole Volume (mL)	103 (92–113)	108 (95–120)	95 (86–104)	0.27
Left Ventricular End-systole Volume (mL)	58 (50–66)**	52 (42–62)	46 [40–53]	0.07
Left Ventricular Ejection Fraction (%)	45 [41–49]**	54 (50–58)	53 (50–56)	*<0.01
Left Ventricular Wall Motion Score Index	2.00 (1.83–2.17)	1.80 (1.51–2.10)	1.78 (1.61–1.96)	0.18
Left Ventricular Global Longitudinal Strain (%)	-11.88 (-12.99- -10.78)*	-13.1 (-14.55- -11.66)	-13.87 (-15.03- -12.72)	*0.04
Left Ventricular Outflow Tract Stroke Volume (mL)	53 (48–59)**	57 (49–64)	64 (57–71)	*0.04
e’ Septal	5.48 (5.04–5.91)**	6.12 (5.54–6.69)	6.50 (5.86–7.15)	*0.02
e’ Lateral	7.56 (6.85–8.27)	8.54 (7.64–9.44)	8.03 (7.09–8.97)	0.25
Mitral Valve E Wave (cm/s)	82 (75–90)	83 (72–93)	87 (78–95)	0.74
MV-A (cm/s)	81 (74–89)	79 (69–88)	86 (77–94)	0.52
E/A Ratio	1.06 (0.93–1.19)	1.05 (0.90–1.21)	1.06 (0.89–1.22)	0.99
E/e’ Septal	16.51 (14.45–18.58)	14.64 (11.60-17.67)	14.28 (12.43–16.12)	0.30
E/e’ Lateral	12.10 (10.48–13.72)	10.91 (8.86–12.96)	11.58 (9.83–13.34)	0.63
Left Atrial End-systolic Volume Index (mL/m^2^)	26 [24–29]	23 [20–26]	25 [21–29]	0.39

* = *P* ≤ 0.05 when comparing all groups

** = *P* ≤ 0.05 when comparing Cluster A vs. Cluster C

### Supervised machine learning classifier for phenogroups

**Fig. 4:**
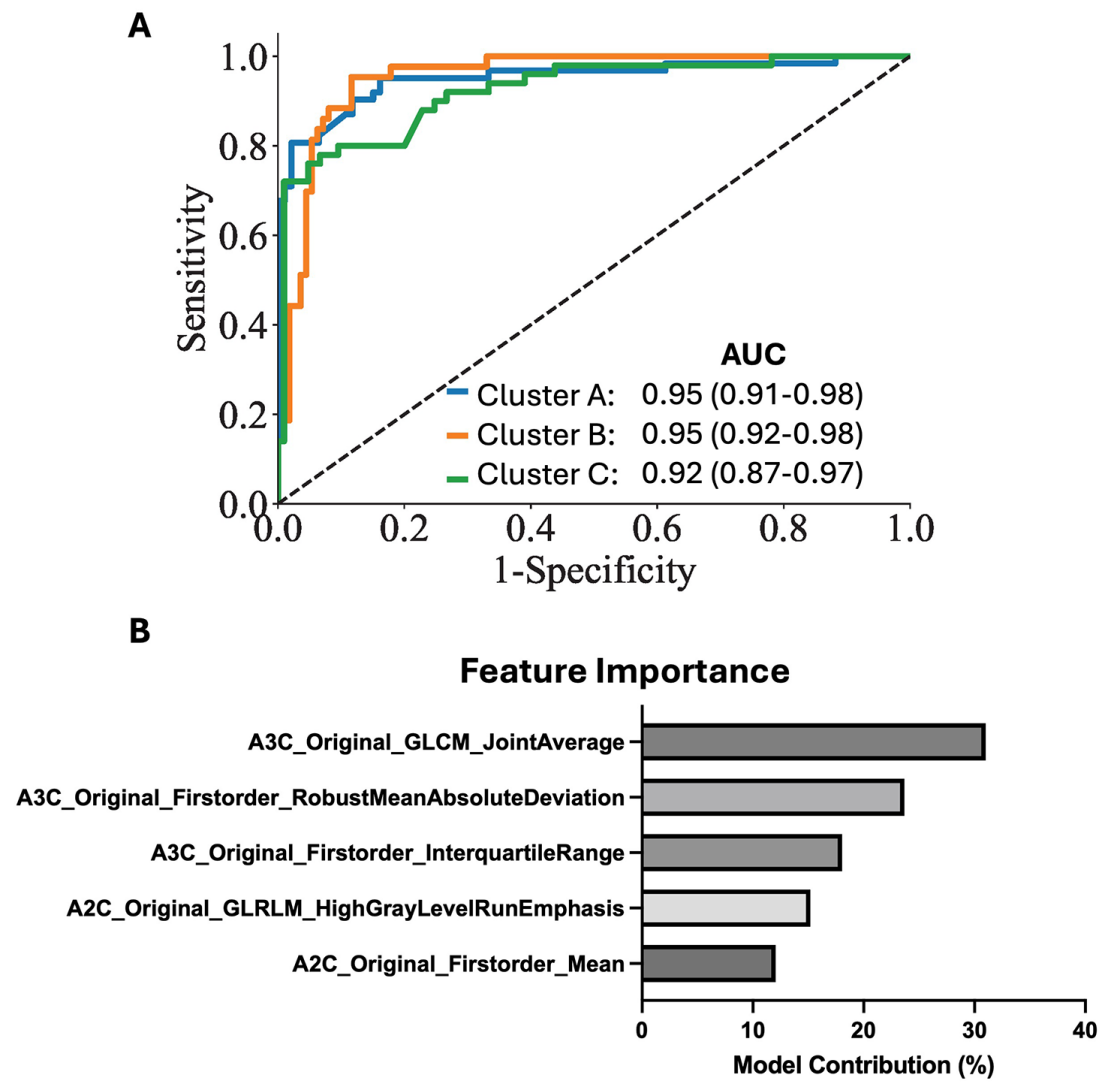
Supervised Machine Learning Classifier. (**A**) Prediction of phenogroup labels on the internal validation dataset using only ultrasomics **(B**) The top five features contributing to model development for the supervised machine learning classifier

With only ultrasomics features, the phenogroup labels were predicted for Cluster A (ROC AUC: 0.95), Cluster B (ROC AUC: 0.95), and Cluster C (ROC AUC: 0.92) (Fig. 4A). When looking at the features contributing to the model, there was a mix of texture-based features and first order features (Fig. 4B). Prediction probabilities were generated for the internal validation dataset based on the supervised classifier; these probabilities were used in subsequent analyses for risk prediction.

### Outcome prediction in the internal and external patient groups

**Fig. 5:**
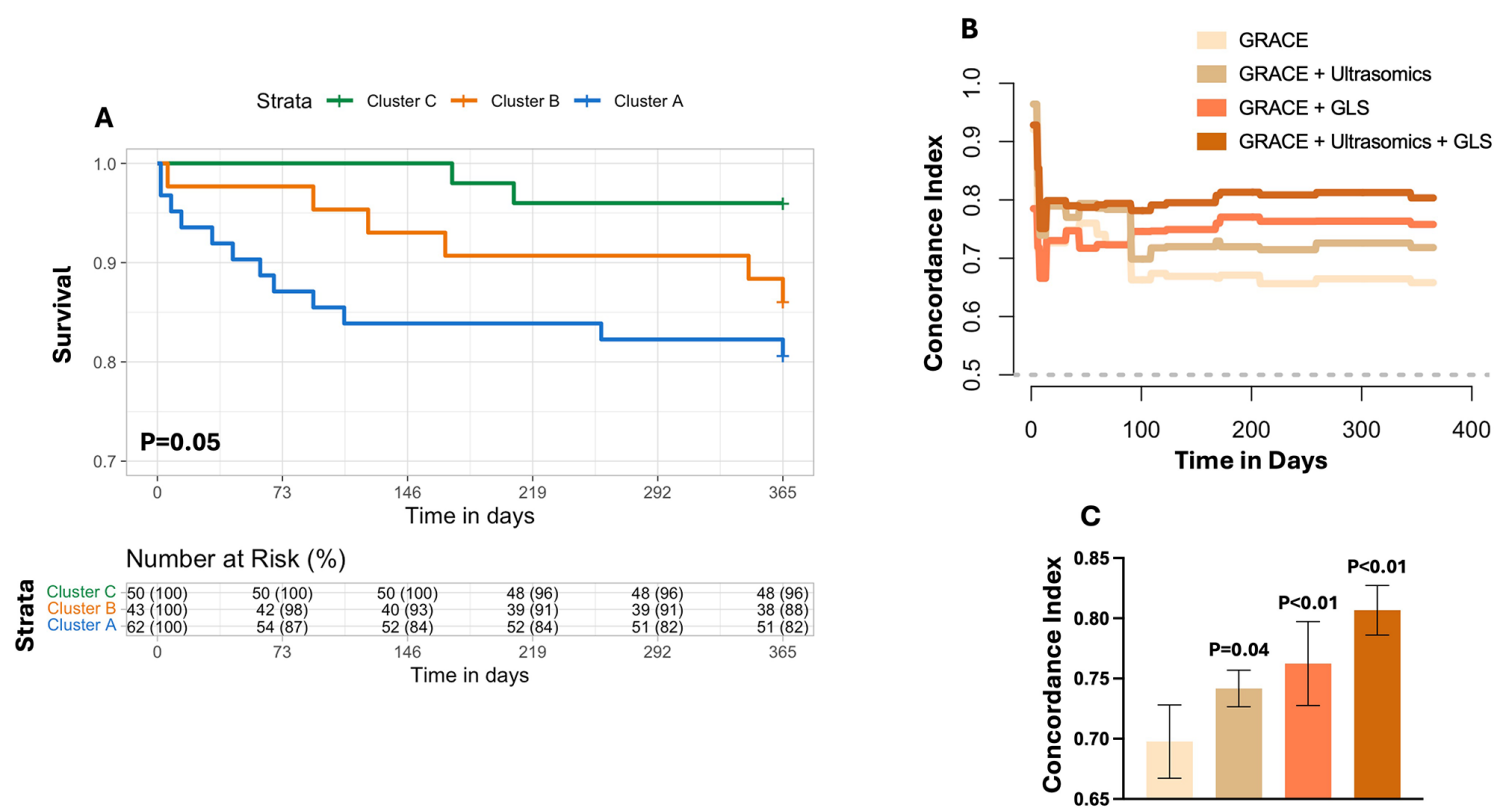
Performance of Phenogroups in Assessing All-Cause Mortality. (**A**) Kaplan Meyer curve and stratified risk categories for patients in phenogroups Cluster A, Cluster B, and Cluster C. (**B**) Time-to-event Concordance Index (C-Index) for groups (**1**) GRACE 2.0 score alone, (**2**) GRACE + Cluster A, (**3**) GRACE + left ventricular global longitudinal strain (GLS), and (**4**) using all three variables through CoxPH regression. (**C**) Incremental value of ultrasomics features (i.e., Cluster A) in predicting all-cause mortality, over the 1-year follow-up period. GRACE = Global Registry of Acute Coronary Events

Using mortality at one year, survival analysis revealed that patients assigned to Cluster A had a significant increase in mortality compared to Cluster C (log rank, *P* = 0.05) (Fig. 5A). We further wanted to further understand if the phenogroups, represented by changes in ultrasomics, had incremental value when predicting mortality. The concordance index was calculated for our four groups of variables: (A) GRACE 2.0 score alone, (B) GRACE + Cluster A, (C) GRACE + LV global longitudinal strain, and (D) using all three variables together (Fig. 5B). When examining GRACE scoring combined with ultrasomics (Concordance: 0.74 vs. 0.70, *P* = 0.04) and further adding LV GLS (Concordance: 0.81 vs. 0.70, *P* < 0.01), an increase in prediction of all-cause mortality is shown beyond that of the GRACE 2.0 score alone, respectively (Fig. 5C).

The developed supervised model was further applied to the external participants to assign phenogroup labels (i.e., Cluster A, B, and C). The batch prediction of the external dataset (*n* = 42 presenting with STEMI) labeled participants into Cluster A (*n* = 11), Cluster B (*n* = 23), and Cluster C (*n* = 8) (Table 5). Patients in Cluster A had a higher percentage of LV identified as “at risk” (60% vs. 37%, *P* = 0.04) at 5 days post AMI, compared to Cluster C. Moreover, patients in the Cluster A phenogroup had a higher proportion of large infarcts (> 10% of LV mass) at 30 days following AMI (0.91 vs. 0.25, *P* < 0.01), when compared to Cluster C.

**Table 5:** Patient demographics of the External Validation Group for Predicted Ultrasomics Phenogroups. Class labels were generated for the external hold out dataset (i.e., the prospective, multicenter, randomized DTU-STEMI pilot trial dataset). Labels were applied based solely on ultrasomics features from the A4C, A3C, and A2C echocardiogram views. Data are considered statistically significant if *P* ≤ 0.05, denoted by * and bolded text. BMI = body mass index, CHF = congestive heart failure, COPD = chronic obstructive pulmonary disease, CAD = coronary artery disease, CKD = chronic kidney disease, MACE = major adverse cardiac events, LV = left ventricular

External Validation - Patient Demographics in Predicted Ultrasomics Phenogroups				
Variable	Cluster A (High Risk) (*n* = 11)	Cluster B (*n* = 23)	Cluster C (Low Risk) (*n* = 8)	*P*-Value
Age (years)	56.82 (48.07–65.57)	58.26 (53.72–62.8)	62.88 (54.99–70.76)	0.48
Sex (Male)	9 (81.82%)	18 (78.26%)	5 (62.5%)	0.60
Race/Ethnicity Caucasian Asian American Hispanic American Black/African American	8 (72.73%) 1 (9.09%) 0 (0%) 1 (9.09%)	17 (73.91%) 3 (13.04%) 1 (4.55%) 3 (13.04%)	6 (75.00%) 0 (0%) 0 (0%) 2 (25.00%)	0.99 0.58 0.66 0.62
BMI (kg/m^2^)	30.08 (25.73–34.42)	31.61 (27.35–35.88)	25.61 (21.55–29.67)	0.23
Systolic Blood Pressure (mmHg)	148 (129–167)	158 (143–173)	144 (132–157)	0.44
Diastolic Blood Pressure (mmHg)	93 (81–104)	91 (84–99)	87 (75–99)	0.71
Heart Rate (per minute)	85 (77–93)	91 (81–101)	81 (66–96)	0.45
Left Ventricular Ejection Fraction (%)	36 [27–45]	37 [31–43]	44 [37–51]	0.41
Mitral Valve E Wave (cm/s)	74 (58–91)	77 (69-0.85)	74 (60–89)	0.93
Mitral Valve A Wave (cm/s)	72 (62–83)	69 (59–78)	74 (64–85)	0.71
E/A Ratio	1.07 (0.78–1.37)	1.20 (0.98–1.42)	1.02 (0.79–1.24)	0.55
History of COPD	0 (0%)	0 (0%)	0 (0%)	0.99
History of CAD	1 (9.09%)	1 (4.35%)	1 (12.50%)	0.73
History of CKD	0 (0%)	0 (0%)	0 (0%)	-
History of Diabetes Mellitus	3 (27.27%)	4 (17.39%)	0 (0%)	0.30
History of Hyperlipidemia	7 (63.64%)	9 (39.13%)	4 (50.00%)	0.42
Prior Stroke	1 (9.09%)	0 (0%)	0 (0%)	0.25
MACE − 30 Days	0 (0%)	2 (8.70%)	0 (0%)	0.44
Cardiovascular Death − 30 Days	0 (0%)	0 (0%)	0 (0%)	-
All Cause Mortality − 30 Days	0 (0%)	0 (0%)	0 (0%)	-
Infarct Size (%) of Area at Risk − 5 Days	60 (52–68)**	46 (37–56)	37 (18–56)	0.06
Acute Volume of Infarct Size (mL) − 5 Days	43 [32–54]	31 [20–42]	21 (-4.12-46)	0.17
Acute Infarct Size (%) of LV Mass − 5 Days	23 [17–29]	17 [11–23]	12 (-0.53-25)	0.24
Acute Infarct Size > 10% of LV Mass − 5 Days	9 (82.00%)	14 (61%)	3 (38.00%)	0.07
Acute Volume of Infarct Size (mL) − 30 Days	28 [21–36]	23 [14–32]	14 (-3.10-31)	0.25
Acute Infarct Size (%) of LV Mass − 30 Days	18 [13–22]	14 (8.73-19)	9.23 (-1.31-20)	0.27
Acute Infarct Size > 10% of LV Mass − 30 Days	10 (91.00%)	11 (48.00%)	2 (25.00%)	*<0.01

* = *P* ≤ 0.05 when comparing all groups

** = *P* ≤ 0.05 when comparing Cluster A vs. Cluster C

## Discussion

Properties of pathological changes within the myocardial microstructure influence ultrasound signal intensity distributions [29]. Unlike information obtained indirectly (i.e., clinical risk factors, ECG, and biomarkers), specific analyzable trends in ultrasound texture information may have added insights into causal pathways that result in disease and clinical presentation. Integrating myocardial texture analysis (i.e., ultrasomics) with clinical data can provide a rich opportunity to develop machine learning models to predict adverse cardiac events following AMI, as ultrasomics can identify cellular changes in the myocardium [29, 36]. To this end we provide a proof-of-concept application of ultrasomics (i.e., cardiac ultrasound radiomics) in risk stratifying AMI patients. Three AMI phenogroups were identified according to ultrasound texture features with patients in phenogroup A having the worst prognosis. Phenogroup A showed incremental and independent information over GRACE 2.0 for predicting 1-year mortality after AMI. Using a cluster-then-predict framework we utilized an external hold out dataset for phenogroup prediction in which phenogroup A had large proportion of patients with moderate or large infarcts.

While classic supervised learning approaches require larger datasets, the cluster-then-predict methodology has the advantage of reducing bias, such as overfitting, when risk stratifying patients [37–39]. Moreover this approach reduces prediction errors [40] and shows robust performance with echo-related data [41–44]. Radiomics, deep learning features, 2D-echocardiography, demographic/clinical (e.g., age, sex, race, BSA, BMI, comorbidities, family history, etc.), laboratory, and biomarker data can further be added to incrementally increase the risk-stratification of these phenogroups. Our group has previously utilized TDA to create patient similarity networks to identify aortic stenosis [45], diastolic dysfunction [46–48], and heart failure [49, 50]. In aortic stenosis, by creating patient phenogroups for mild and severe aortic stenosis, the “high-risk” severe aortic stenosis phenogroup was associated with increased risk of balloon valvuloplasty, and valve replacement [45]. Specifically, as shown in this study, the phenotypic groups from TDA (or unsupervised machine learning, PCA clustering, etc.) can serve as class labels for developing supervised algorithms. This technique, first clustering and then predicting using supervised machine-learning models, can result in stronger associations with clinical outcomes by increasing the number of events (i.e., phenogroup clusters) and reduce class imbalance.

Current risk stratification tools for AMI, such as the GRACE Score, reduce mortality rates compared to standard strategies [51, 52] but, with the use of current AI applications, it is possible to characterize more patients at-risk for morbidity and mortality by combining information from clinical, laboratory, imaging, and other features. Risk stratification tools can be benchmarked using AUC and C-Index as metrics, with values ranging from 0.6 to 0.7 having limited clinical value, whereas those between 0.7 and 0.8, 0.8–0.9, and > 0.9 considered to have fair, good, and excellent discrimination [53–55], respectively. The GRACE model has shown performances ranging from 0.65 to 0.8 (C-Index) [9], with our current study reporting a performance of 0.70, utilizing the GRACE 2.0 score, which is within the reported variation of the model. We also showed how the C-Index improved when using ultrasomics features (0.74) and in combination with LV functional parameters (0.81). As this is a feasibility study, future work should harness these non-clinical markers (such as ultrasomics and LV functional information) in larger, multicenter studies to create new risk stratification tools for the prediction of AMI.

We note several limitations to the current investigation. (1) The cohort sizes in the internal and external validations sets are relatively small (*n* = 155 and *n* = 42, respectively). While this patient groups are small, we highlight how the cluster-then-predict methodology is better adapted to smaller datasets and can help provide a framework for other investigations where small cohort sizes are present (i.e., rare diseases, underrepresented minorities, limited resources for data collection, etc.). Though, as the external validation cohort (*n* = 42) is further stratified into smaller clustered groups within our analysis, the generalizability of these results is limited and requires a larger external validation group in the future to assess the robustness of the current findings. (2) The outcome of interest, all-cause mortality at 1 year, was only represented in 20 of 155 patients. Because of the low number of events, we used univariate analysis to screen for features to provide in the adjusted model while avoid issues with overfitting in the survival model. Nevertheless, we noted the incremental value of radiomics over conventional scores like GRACE 2.0 and several echocardiographic parameters like ejection fraction, LV end-systolic volume and global longitudinal strain. Future work with larger sample size and a greater number of events would allow develop of robust multivariable models using radiomics, clinical and conventional echocardiographic features. (3) The use of TDA, and other unsupervised learning approaches, can be subjective in the number of clusters defined. In the current study, we highlight three unique phenogroups. While we could have altered the parameters to include more or less numbers of phenogroups, the main constraint on the Mapper algorithm that we wanted to maintain was a low percent overlap between groups (i.e., reducing the similarities of phenogroups and ultimately providing clearer boundaries between those with “high” and “low” risk).

## Conclusions

In summary, we utilize an echocardiography-derived approach to measure ultrasomics and identify phenogroups among patients presenting with AMI. Through TDA, three distinct phenogroups (Clusters A, B, and C) were delineated, with Cluster A representing a “high-risk” group, Cluster B an “intermediate-risk” group, and Cluster C a “low-risk” group. These phenogroups demonstrated significant differences in clinical outcomes, particularly in terms of all-cause mortality at 1 year. Logistic regression and supervised machine learning further validate the predictive power of these phenogroups, showing their potential utility in clinical risk stratification. Moreover, application of the developed model to an external dataset highlighted the robustness of these phenogroups in predicting cardiac magnetic resonance (CMR) findings such as infarct size, providing valuable insights for personalized patient management and prognostication in AMI.

## Electronic supplementary material

Below is the link to the electronic supplementary material.

Supplementary Material 1

## Data Availability

All code is made freely available on our GitHub repository https://github.com/qahathaway/AMI_Phenogroups. All data is available by reasonable request.

## Abbreviations

AMI: Acute Myocardial Infarction
GRACE: Global Registry of Acute Coronary Events
TDA: Topological Data Analysis
LV: Left Ventricle
CMR: Cardiac Magnetic Resonance
NSTEMI: Non-ST-Elevation Myocardial Infarction
STEMI: ST-Elevation Myocardial Infarction
AI: Artificial Intelligence
CNNs: Convolutional Neural Networks
ROI: Region of Interest

## References

[1] Salari N, Morddarvanjoghi F, Abdolmaleki A, Rasoulpoor S, Khaleghi AA, Hezarkhani LA, et al. The global prevalence of myocardial infarction: a systematic review and meta-analysis. BMC Cardiovasc Disord. 2023;23(1):206. 37087452 10.1186/s12872-023-03231-w PMC10122825

[2] Bishu KG, Lekoubou A, Kirkland E, Schumann SO, Schreiner A, Heincelman M, et al. Estimating the Economic Burden of Acute myocardial infarction in the US: 12 Year National Data. Am J Med Sci. 2020;359(5):257–65. 32265010 10.1016/j.amjms.2020.02.004

[3] Tsao CW, Aday AW, Almarzooq ZI, Alonso A, Beaton AZ, Bittencourt MS, et al. Heart Disease and Stroke Statistics—2022 update: a Report from the American Heart Association. Circulation. 2022;145(8):e153–639. 35078371 10.1161/CIR.0000000000001052

[4] Fox KAA, Dabbous OH, Goldberg RJ, Pieper KS, Eagle KA, Van de Werf F, et al. Prediction of risk of death and myocardial infarction in the six months after presentation with acute coronary syndrome: prospective multinational observational study (GRACE). BMJ. 2006;333(7578):1091. 17032691 10.1136/bmj.38985.646481.55 PMC1661748

[5] Eagle KA, Lim MJ, Dabbous OH, Pieper KS, Goldberg RJ, Van De Werf F, et al. A validated prediction model for all forms of Acute Coronary Syndrome. JAMA. 2004;291(22):2727. 15187054 10.1001/jama.291.22.2727

[6] Fox KA, Fitzgerald G, Puymirat E, Huang W, Carruthers K, Simon T, et al. Should patients with acute coronary disease be stratified for management according to their risk? Derivation, external validation and outcomes using the updated GRACE risk score. BMJ Open. 2014;4(2):e004425. 24561498 10.1136/bmjopen-2013-004425 PMC3931985

[7] Collet J-P, Thiele H, Barbato E, Barthélémy O, Bauersachs J, Bhatt DL, et al. 2020 ESC guidelines for the management of acute coronary syndromes in patients presenting without persistent ST-segment elevation: the Task Force for the management of acute coronary syndromes in patients presenting without persistent ST-segment elevation of the European Society of Cardiology (ESC). Eur Heart J. 2020;42(14):1289–367. 10.1093/eurheartj/ehaa575 32860058

[8] Gulati M, Levy PD, Mukherjee D, Amsterdam E, Bhatt DL, Birtcher KK, AHA/ACC/ASE/CHEST/, SAEM/SCCT/SCMR Guideline for the Evaluation and Diagnosis of Chest Pain. Executive Summary: A Report of the American College of Cardiology/American Heart Association Joint Committee on Clinical Practice Guidelines. Circulation. 2021;144(22):e336-e67. 10.1161/CIR.0000000000001030 34709928

[9] D’Ascenzo F, Biondi-Zoccai G, Moretti C, Bollati M, Omedè P, Sciuto F, et al. TIMI, GRACE and alternative risk scores in Acute Coronary syndromes: a meta-analysis of 40 derivation studies on 216,552 patients and of 42 validation studies on 31,625 patients. Contemp Clin Trials. 2012;33(3):507–14. 22265976 10.1016/j.cct.2012.01.001

[10] Rajpurkar P, Chen E, Banerjee O, Topol EJ. AI in health and medicine. Nat Med. 2022;28(1):31–8. 35058619 10.1038/s41591-021-01614-0

[11] Koh D-M, Papanikolaou N, Bick U, Illing R, Kahn CE, Kalpathi-Cramer J, et al. Artificial intelligence and machine learning in cancer imaging. Commun Med. 2022;2(1):133. 36310650 10.1038/s43856-022-00199-0 PMC9613681

[12] Gulshan V, Peng L, Coram M, Stumpe MC, Wu D, Narayanaswamy A, et al. Development and validation of a deep learning algorithm for detection of Diabetic Retinopathy in Retinal Fundus photographs. JAMA. 2016;316(22):2402–10. 27898976 10.1001/jama.2016.17216

[13] Huynh E, Hosny A, Guthier C, Bitterman DS, Petit SF, Haas-Kogan DA, et al. Artificial intelligence in radiation oncology. Nat Rev Clin Oncol. 2020;17(12):771–81. 32843739 10.1038/s41571-020-0417-8

[14] McKinney SM, Sieniek M, Godbole V, Godwin J, Antropova N, Ashrafian H, et al. International evaluation of an AI system for breast cancer screening. Nature. 2020;577(7788):89–94. 31894144 10.1038/s41586-019-1799-6

[15] Ardila D, Kiraly AP, Bharadwaj S, Choi B, Reicher JJ, Peng L, et al. End-to-end lung cancer screening with three-dimensional deep learning on low-dose chest computed tomography. Nat Med. 2019;25(6):954–61. 31110349 10.1038/s41591-019-0447-x

[16] Zhou D, Tian F, Tian X, Sun L, Huang X, Zhao F, et al. Diagnostic evaluation of a deep learning model for optical diagnosis of colorectal cancer. Nat Commun. 2020;11(1):2961. 32528084 10.1038/s41467-020-16777-6 PMC7289893

[17] Lambin P, Leijenaar RTH, Deist TM, Peerlings J, de Jong EEC, van Timmeren J, et al. Radiomics: the bridge between medical imaging and personalized medicine. Nat Reviews Clin Oncol. 2017;14(12):749–62. 10.1038/nrclinonc.2017.141 28975929

[18] Cho H-h, Lee HY, Kim E, Lee G, Kim J, Kwon J, et al. Radiomics-guided deep neural networks stratify lung adenocarcinoma prognosis from CT scans. Commun Biology. 2021;4(1):1286. 10.1038/s42003-021-02814-7 PMC8590002 34773070

[19] Wang Y, Yue W, Li X, Liu S, Guo L, Xu H, et al. Comparison study of Radiomics and Deep Learning-based methods for thyroid nodules classification using Ultrasound images. IEEE Access. 2020;8:52010–7. 10.1109/ACCESS.2020.2980290

[20] Afshar P, Mohammadi A, Plataniotis KN, Oikonomou A, Benali H. From handcrafted to Deep-Learning-Based Cancer Radiomics: challenges and opportunities. IEEE Signal Process Mag. 2019;36(4):132–60. 10.1109/MSP.2019.2900993

[21] Hunter B, Chen M, Ratnakumar P, Alemu E, Logan A, Linton-Reid K, et al. A radiomics-based decision support tool improves lung cancer diagnosis in combination with the Herder score in large lung nodules. EBioMedicine. 2022;86:104344. 36370635 10.1016/j.ebiom.2022.104344 PMC9664396

[22] Chazal F, Michel B. An introduction to Topological Data Analysis: fundamental and practical aspects for data scientists. Front Artif Intell. 2021;4:667963. 34661095 10.3389/frai.2021.667963 PMC8511823

[23] Stone GW, Selker HP, Thiele H, Patel MR, Udelson JE, Ohman EM, et al. Relationship between infarct size and outcomes following primary PCI: patient-level analysis from 10 randomized trials. J Am Coll Cardiol. 2016;67(14):1674–83. 27056772 10.1016/j.jacc.2016.01.069

[24] Kapur NK, Alkhouli MA, DeMartini TJ, Faraz H, George ZH, Goodwin MJ, et al. Unloading the left ventricle before reperfusion in patients with Anterior ST-Segment-Elevation myocardial infarction. Circulation. 2019;139(3):337–46. 30586728 10.1161/CIRCULATIONAHA.118.038269

[25] Thygesen K, Alpert JS, Jaffe AS, Simoons ML, Chaitman BR, White HD, et al. Third universal definition of myocardial infarction. Eur Heart J. 2012;33(20):2551–67. 22922414 10.1093/eurheartj/ehs184

[26] Al-Hussaini A, Abdelaty A, Gulsin GS, Arnold JR, Garcia-Guimaraes M, Premawardhana D, et al. Chronic infarct size after spontaneous coronary artery dissection: implications for pathophysiology and clinical management. Eur Heart J. 2020;41(23):2197–205. 31898721 10.1093/eurheartj/ehz895 PMC7299635

[27] Krljanac G, Apostolovic S, Polovina M, Maksimovic R, Nedeljkovic Arsenovic O, Dordevic N, et al. Differences in left ventricular myocardial function and infarct size in female patients with ST elevation myocardial infarction and spontaneous coronary artery dissection. Front Cardiovasc Med. 2023;10:1280605. 38259320 10.3389/fcvm.2023.1280605 PMC10800883

[28] Zhang J, Gajjala S, Agrawal P, Tison GH, Hallock LA, Beussink-Nelson L, et al. Fully automated Echocardiogram Interpretation in Clinical Practice. Circulation. 2018;138(16):1623–35. 30354459 10.1161/CIRCULATIONAHA.118.034338 PMC6200386

[29] Hathaway QA, Yanamala N, Siva NK, Adjeroh DA, Hollander JM, Sengupta PP. Ultrasonic texture features for assessing Cardiac Remodeling and Dysfunction. J Am Coll Cardiol. 2022;80(23):2187–201. 36456049 10.1016/j.jacc.2022.09.036

[30] van Griethuysen JJM, Fedorov A, Parmar C, Hosny A, Aucoin N, Narayan V, et al. Computational Radiomics System to Decode the Radiographic phenotype. Cancer Res. 2017;77(21):e104–7. 29092951 10.1158/0008-5472.CAN-17-0339 PMC5672828

[31] Yaniv Z, Lowekamp BC, Johnson HJ, Beare R. SimpleITK Image-Analysis notebooks: a Collaborative Environment for Education and Reproducible Research. J Digit Imaging. 2018;31(3):290–303. 29181613 10.1007/s10278-017-0037-8 PMC5959828

[32] Walsh K, Voineagu MA, Vafaee F, Voineagu I. TDAview: an online visualization tool for topological data analysis. Bioinformatics. 2020;36(18):4805–9. 32614445 10.1093/bioinformatics/btaa600

[33] Singh G, Mémoli F, Carlsson G. Topological Methods for the Analysis of High Dimensional Data Sets and 3D Object Recognition. In: Botsch M, Pajarola R, editors. Eurographics Symposium on Point-Based Graphics (2007); Prague: The Eurographics Association; 2007.

[34] Therneau TM. A Package for Survival Analysis in R. 2022. p. R package version 3.4-0.

[35] Mogensen UB, Ishwaran H, Gerds TA. Evaluating Random Forests for Survival Analysis using Prediction Error curves. J Stat Softw. 2012;50(11):1–23. 25317082 10.18637/jss.v050.i11 PMC4194196

[36] Marwick TH. Assessment of Myocardial texture: the Next Frontier in echocardiographic quantification. J Am Coll Cardiol. 2022;80(23):2202–4. 36456050 10.1016/j.jacc.2022.10.003

[37] Ma EY, Kim JW, Lee Y, Cho SW, Kim H, Kim JK. Combined unsupervised-supervised machine learning for phenotyping complex diseases with its application to obstructive sleep apnea. Sci Rep. 2021;11(1):4457. 33627761 10.1038/s41598-021-84003-4 PMC7904925

[38] Soni R, Mathai KJ. An innovative ‘Cluster-then-predict’ Approach for Improved sentiment prediction. In: Choudhary R, Mandal J, Auluck N, Nagarajaram H, editors. Advanced Computing and Communication Technologies. Singapore: Springer; 2016. pp. 131–40.

[39] Yuill W, Kunz H. Using machine learning to Improve Personalised Prediction: A Data-Driven Approach to Segment and Stratify populations for Healthcare. Stud Health Technol Inf. 2022;289:29–32. 10.3233/SHTI210851 35062084

[40] Trivedi S, Pardos ZA, Heffernan NT. The utility of clustering in prediction tasks. arXiv Preprint arXiv:150906163. 2015.

[41] Kagiyama N, Shrestha S, Cho JS, Khalil M, Singh Y, Challa A, et al. A low-cost texture-based pipeline for predicting myocardial tissue remodeling and fibrosis using cardiac ultrasound. EBioMedicine. 2020;54:102726. 32268274 10.1016/j.ebiom.2020.102726 PMC7139137

[42] Tokodi M, Shrestha S, Bianco C, Kagiyama N, Casaclang-Verzosa G, Narula J, et al. Interpatient similarities in cardiac function: a platform for personalized cardiovascular medicine. Cardiovasc Imaging. 2020;13(5):1119–32. 10.1016/j.jcmg.2019.12.018 PMC7556337 32199835

[43] Pandey A, Kagiyama N, Yanamala N, Segar MW, Cho JS, Tokodi M, et al. Deep-learning models for the echocardiographic assessment of diastolic dysfunction. Cardiovasc Imaging. 2021;14(10):1887–900. 10.1016/j.jcmg.2021.04.010 34023263

[44] Sengupta PP, Shrestha S, Kagiyama N, Hamirani Y, Kulkarni H, Yanamala N, et al. A machine-learning Framework to identify distinct phenotypes of aortic stenosis severity. JACC Cardiovasc Imaging. 2021;14(9):1707–20. 34023273 10.1016/j.jcmg.2021.03.020 PMC8434951

[45] Casaclang-Verzosa G, Shrestha S, Khalil MJ, Cho JS, Tokodi M, Balla S, et al. Network Tomography for understanding phenotypic presentations in aortic stenosis. JACC Cardiovasc Imaging. 2019;12(2):236–48. 30732719 10.1016/j.jcmg.2018.11.025

[46] Pandey A, Kagiyama N, Yanamala N, Segar MW, Cho JS, Tokodi M, et al. Deep-learning models for the echocardiographic Assessment of Diastolic Dysfunction. JACC Cardiovasc Imaging. 2021;14(10):1887–900. 34023263 10.1016/j.jcmg.2021.04.010

[47] Shah R, Tokodi M, Jamthikar A, Bhatti S, Akhabue E, Casaclang-Verzosa G et al. A deep patient-similarity Learning Framework for the Assessment of Diastolic Dysfunction in Elderly patients. Eur Heart J Cardiovasc Imaging. 2024. 10.1093/ehjci/jeae037 38315669

[48] Tokodi M, Shrestha S, Bianco C, Kagiyama N, Casaclang-Verzosa G, Narula J, et al. Interpatient similarities in cardiac function: a platform for personalized Cardiovascular Medicine. JACC Cardiovasc Imaging. 2020;13(5):1119–32. 32199835 10.1016/j.jcmg.2019.12.018 PMC7556337

[49] Cho JS, Shrestha S, Kagiyama N, Hu L, Ghaffar YA, Casaclang-Verzosa G, et al. A network-based Phenomics Approach for discovering patient subtypes from high-throughput Cardiac Imaging Data. JACC Cardiovasc Imaging. 2020;13(8):1655–70. 32762883 10.1016/j.jcmg.2020.02.008

[50] Patel HB, Yanamala N, Patel B, Raina S, Farjo PD, Sunkara S, et al. Electrocardiogram-based machine learning Emulator Model for Predicting Novel Echocardiography-Derived Phenogroups for Cardiac Risk-Stratification: a prospective Multicenter Cohort Study. J Patient Cent Res Rev. 2022;9(2):98–107. 35600228 10.17294/2330-0698.1893 PMC9022713

[51] Hall M, Bebb OJ, Dondo TB, Yan AT, Goodman SG, Bueno H, et al. Guideline-indicated treatments and diagnostics, GRACE risk score, and survival for non-ST elevation myocardial infarction. Eur Heart J. 2018;39(42):3798–806. 30202849 10.1093/eurheartj/ehy517 PMC6220125

[52] van der Sangen NMR, Azzahhafi J, Chan Pin Yin D, Peper J, Rayhi S, Walhout RJ et al. External validation of the GRACE risk score and the risk-treatment paradox in patients with acute coronary syndrome. Open Heart. 2022;9(1). 10.1136/openhrt-2022-001984 PMC8969003 35354660

[53] Ohman EM, Granger CB, Harrington RA, Lee KL. Risk stratification and therapeutic decision making in acute coronary syndromes. JAMA. 2000;284(7):876–8. 10938178 10.1001/jama.284.7.876

[54] Shann F. Are we doing a good job: PRISM, PIM and all that. Intensive Care Med. 2002;28(2):105–7. 11907652 10.1007/s00134-001-1186-1

[55] Solomon LJ. Mortality risk prediction models: methods of assessing discrimination and calibration and what they mean. South Afr J Crit Care. 2022;38(1). 10.7196/SAJCC.2022.v38i1.548 PMC9132075 35634480

