# Supplementary material for: Cardiac ultrasomics for acute myocardial infarction risk stratification and prediction of all-cause mortality: a feasibility study
Source: Echo Res Pract. 2024 Sep 16;11:22. doi: 10.1186/s44156-024-00057-w (PMC11403884; doi:10.1186/s44156-024-00057-w)
Supplement: Supplementary file 1 — Supplementary Material 1 [file 44156_2024_57_MOESM1_ESM.docx]

**Cardiac Ultrasomics for Acute Myocardial Infarction Risk Stratification and Prediction of All-Cause Mortality: A Feasibility Study**

Quincy A. Hathaway, MD, PhD^1,2^*****, Ankush D. Jamthikar, PhD^1^*****, Nivedita Rajiv^1^, Bernard R. Chaitman, MD^3^, Jeffrey L. Carson, MD, MPH^4^, Naveena Yanamala, MS, PhD^1^, Partho P. Sengupta, MD, DM^1^

^1^Division of Cardiovascular Disease and Hypertension, Department of Medicine, Rutgers Robert Wood Johnson Medical School, New Brunswick, NJ, USA.

^2^Department of Radiology, University of Pennsylvania, Philadelphia, PA, USA.

^3^Department of Medicine, St. Louis University School of Medicine, St. Louis, MO, USA

^4^Division of General Internal Medicine, Department of Medicine, Rutgers Robert Wood Johnson Medical School, New Brunswick, NJ, USA

*Running Title: Ultrasonic Texture and Myocardial Infarction Risk Stratification*

*****Provided equal contribution to the work

**Corresponding Author**

Partho P. Sengupta, MD, DM, FACC, FASE

Rutgers Robert Wood Johnson Medical School,

Division of Cardiovascular Disease and Hypertension,

125 Patterson St, New Brunswick, NJ – 08901

Phone: (646) 531-2613

Email: [partho.sengupta@rutgers.edu](mailto:partho.sengupta@rutgers.edu)

**Supplemental Methods**

*Study Population*

In the internal validation dataset, STEMI was classified per the Joint ESC/ACCF/AHA/WHF Task Force (1). Briefly, this included ECG changes revealing 1) new ST-segment elevation in 2 contiguous leads with greater than 0.1 mV in all leads, with the exception of V2 or V3, 2) new ST- segment elevation in leads V2-V3 greater than 0.2 mV (men > 40 years old), 0.25 mV (men < 40 years old), or 0.15 mV (women), 3) Pre-existing left bundle branch block were further evaluated using the Sgarbossa's criteria (2, 3).

For the external validation dataset, participants were recruited from a prospective, multicenter, randomized Door-To-Unload in ST-segment–elevation myocardial infarction (DTU-STEMI) pilot trial (4). We included 42 participants with CMR data in the current study. Briefly, patients were included in the original randomized pilot trial if they 1) were between 21 and 80 years of age and 2) presented with 1-6 hours of chest pain with documented ST-segment elevation of ≥2 mm in ≥2 contiguous anterior leads or ≥4 mm total ST-segment deviation sum in the anterior leads. Patients were excluded if they had prior AMI, coronary artery bypass grafting surgery, out-of-hospital cardiac arrest requiring cardiopulmonary resuscitation, cardiogenic shock, inability to undergo Impella CP insertion, fibrinolysis within 72 hours of presentation, or contraindications to CMR imaging (4). Delayed-enhancement imaging was performed using a 2-dimensional segmented inversion-recovery sequence, 10 minutes after administration of routine extracellular gadolinium contrast (0.15 mmol/kg of body weight). Infarct size was expressed as a percentage of total LV mass. A central core laboratory (Duke Cardiovascular Magnetic Resonance Center, Durham, NC) qualified participating sites, performed quality assessment on the images during the conduct of the study, and manually performed assessment of CMR parameters on deidentified images without knowledge or access to treatment assignment or clinical outcomes.

**Figure S1**


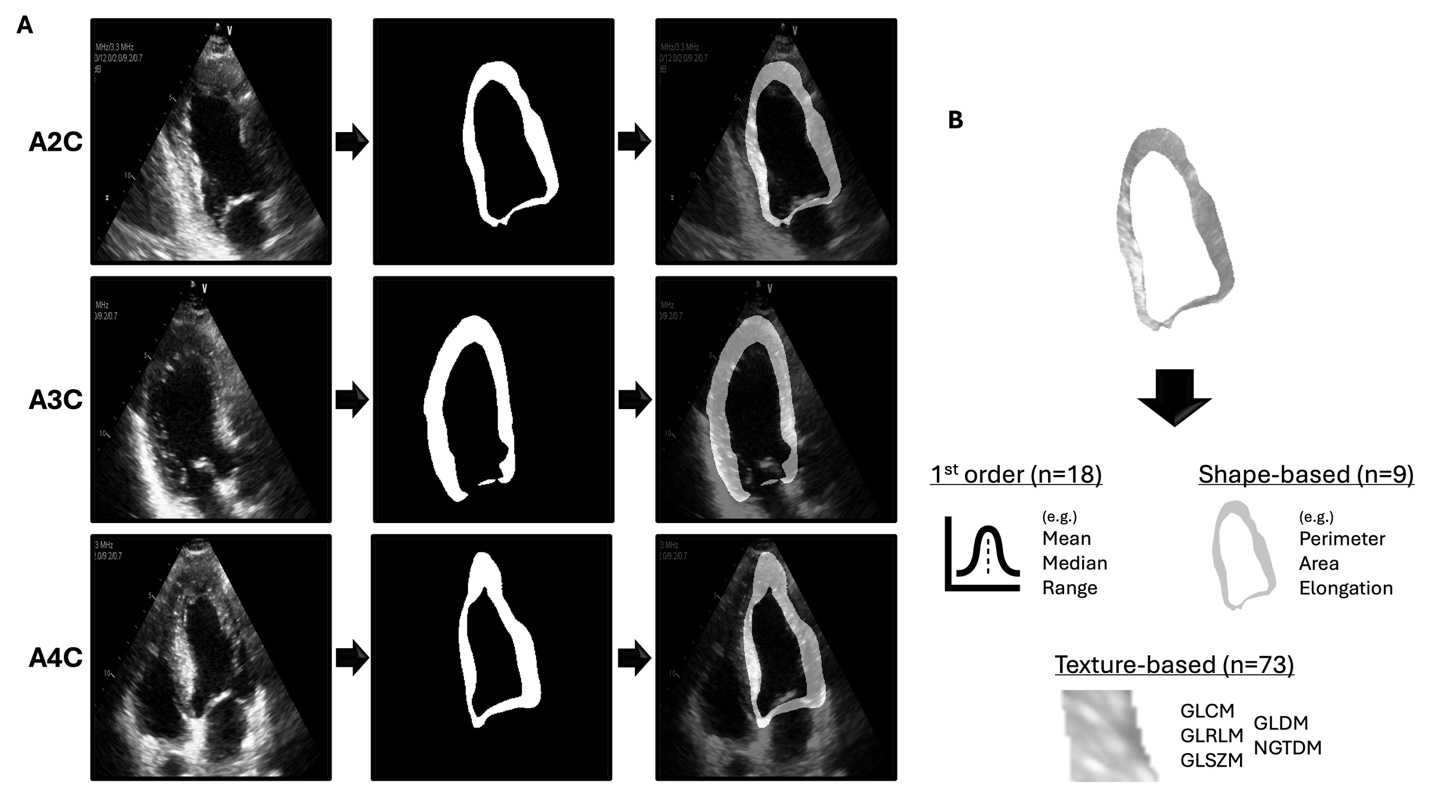


**Figure S1**: **Illustration of Left Ventricular Ultrasomics in Apical 2-Chamber (A2C), Apical 3-Chamber (A3C), and Apical 4-Chamber (A4C).** (**A**) Representative images for region of interest (ROI) placement using semantic segmentation through echocv. (**B**) Example of ultrasomics features extracted from the Python package pyradiomics (v3.0.1), including 1^st^ order (n=18), shape-based (n=9), and texture-based (n=73) features. GLCM = gray-level cooccurrence matrix, GLDM = gray-level difference matrix, NGTDM = Neighborhood gray-tone difference matrix, GLRLM = gray-level run-length, GLSZM = gray-level size zone matrix.

**References**

1. Thygesen K, Alpert JS, Jaffe AS, Simoons ML, Chaitman BR, White HD, et al. Third universal definition of myocardial infarction. Eur Heart J. 2012;33(20):2551-67.

2. Akbar H, Foth C, Kahloon RA, Mountfort S. Acute ST-Elevation Myocardial Infarction. StatPearls. Treasure Island (FL) ineligible companies. Disclosure: Christopher Foth declares no relevant financial relationships with ineligible companies. Disclosure: Rehan Kahloon declares no relevant financial relationships with ineligible companies. Disclosure: Steven Mountfort declares no relevant financial relationships with ineligible companies.2024.

3. Smith SW, Dodd KW, Henry TD, Dvorak DM, Pearce LA. Diagnosis of ST-elevation myocardial infarction in the presence of left bundle branch block with the ST-elevation to S-wave ratio in a modified Sgarbossa rule. Ann Emerg Med. 2012;60(6):766-76.

4. Kapur NK, Alkhouli MA, DeMartini TJ, Faraz H, George ZH, Goodwin MJ, et al. Unloading the Left Ventricle Before Reperfusion in Patients With Anterior ST-Segment-Elevation Myocardial Infarction. Circulation. 2019;139(3):337-46.
